# Feasibility analysis of ambulatory surgery in pediatric ureteropelvic junction obstruction

**DOI:** 10.3389/fped.2025.1623031

**Published:** 2025-07-08

**Authors:** Tong Fang, Rugang Lu, Ting Chen

**Affiliations:** Day Operation Ward, Department of Pediatric Surgery, Children's Hospital of Nanjing Medical University, Nanjing, China

**Keywords:** pediatric, ureteropelvic junction obstruction, ambulatory surgery, laparoscopic pyeloplasty, prognosis

## Abstract

**Objective:**

This study aimed to investigate the feasibility and safety of laparoscopic pyeloplasty for infants and children in an ambulatory surgery setting.

**Methods:**

78 children with ureteropelvic junction obstruction (UPJO) admitted to the Department of Urology in Children's Hospital of Nanjing Medical University from 1 January 2023 to 31 July 2024 (the inpatient group) and 74 children with UPJO admitted to the ambulatory ward from 1 January 2023 to 31 July 2024 (the ambulatory group) were retrospectively analyzed. The two groups were compared with respect to operative time, length of hospital stay, hospitalization cost, and postoperative complications.

**Results:**

There was no statistically significant difference between the two groups in terms of age at surgery, gender, ratio of unilateral UPJO, operative time. The hospitalization cost, length of hospital stay, indwelling urinary catheterization duration, and postoperative double J stent removal time in the ambulatory group were all shorter than those in the inpatient group. There was no significant difference between the two groups in postoperative complications such as reoperation, double J stent replacement, urinary tract infection, fever, vomiting and pain.

**Conclusion:**

Ambulatory surgery for pediatric UPJO is safe and effective. It reduces length of hospital stay and hospitalization cost, effectively improves bed utilization and turnover rate, and does not cause an increase in complication rates.

## Introduction

Ambulatory surgery is defined as surgery in which admission and discharge are completed within 24 h. The originator of this important concept was the Scottish pediatric surgeon James H. Nicoll, who performed nearly 9,000 operations between 1899 and 1908 (1). About half of the patients in this study were infants and young children under the age of 3 years, demonstrating the widespread use and importance of the ambulatory surgery model in this particular group, and Nicoll's study not only pioneered the development of ambulatory surgery, but also provided important practical and theoretical support for future surgical procedures for infants and children. The advantages of ambulatory surgery include shorter hospital stay, reduced nosocomial infections, reduced psychological stress in children and reduced medical costs (2). In recent years, ambulatory surgery for children has flourished in China, but the development of performing complex surgeries in an ambulatory setting is still in its infancy (3).

Pediatric ureteropelvic junction obstruction (UPJO) is a malformation of the urinary system that refers to the dilatation of the renal collecting system detected by screening methods such as ultrasound during the prenatal and early postnatal period (4, 5). This condition may lead to impairment of kidney function. Pyeloplasty can be performed by open or minimally invasive surgery which include laparoscopic or robot-assisted laparoscopic pyeloplasty. The minimally invasive surgery has the advantage of being safe, reliable and less invasive than the open procedure, but requires extensive surgical experience on the part of the operator (6). Children's Hospital of Toronto University performed pyeloplasty in the ambulatory ward from 2008 to 2020 (7). Compared with inpatient surgery, the rate of emergency room visits 30 days after ambulatory surgery was higher, while the readmission and reoperation rates were not statistically significant, which suggests that UPJO is safe and feasible in ambulatory wards.

Children's Hospital of Nanjing Medical University has pioneered ambulatory surgery for UPJO since 2023. In this study, we retrospectively analyze the perioperative and 6-month postoperative follow-up data of children with UPJO admitted to ambulatory wards and urology wards to investigate the feasibility and safety of laparoscopic pyeloplasty for infants and children in an ambulatory surgery setting.

## Methods

### Patients

All children under 18 years of age who underwent laparoscopic pyeloplasty at the Children's Hospital of Nanjing Medical University between January 1, 2023, and July 31, 2024, were retrospectively reviewed. Due to regional healthcare resource constraints, patient allocation was non-randomized and primarily based on geographic proximity to the hospital. Patients residing <100 km were assigned to the ambulatory group (*n* = 74), while those ≥100 km received inpatient care (*n* = 78). We acknowledge this may introduce selection bias, as urban populations may differ from rural populations in socioeconomic status, emergency care access, and family support systems. To mitigate confounding, all patients met identical clinical inclusion criteria, and surgeries were performed by the same team using standardized protocols.

The inclusion criteria were as follows: (1) patients with normal development and no physical deformity; (2) preoperative diagnosis of UPJO through imaging examination, which meets the diagnostic criteria of UPJO (8); (3) patients assessed through surgical anesthesia; (4) patients' families agreed to receive follow-up; (5) Meeting the indications for pyeloplasty: (a) ultrasonography suggests that the anteroposterior diameter of the renal pelvis is >30 mm; (b) the anteroposterior diameter of the renal pelvis is >20 mm, accompanied by dilatation of the renal calyces; (c) hydronephrosis leads to a fractional renal function of less than 40%; (d) a progressive decline in renal function (decline value >10%) during follow-up; (e) a progressive increase in hydronephrosis (increase value >10 mm) during follow-up; and (f) Symptomatic UPJO with recurrent urinary tract infection, loin pain, and/or haematuria; (g) Diuretic renal nuclear scanning suggesting obstruction with t1/2 > 20 min.

Exclusion criteria: (1) respiratory infections within one week; (2) urinary tract infections and unresolved high fever within one week; (3) patients with coagulation disorders or taking anticoagulant medications; (4) patients with severe comorbidities and other systemic diseases.

The study was approved by the Ethics Committee of Children's Hospital of Nanjing Medical University and was carried out in compliance with the International Ethical Guidelines for Research Involving Human Subjects, as outlined in the Declaration of Helsinki. Informed consent was waived because the research utilized anonymized clinical data collected between 2023 and 2024, and all personally identifiable information had been removed prior to analysis.

### Surgical approach

The inpatient group received general anesthesia with endotracheal intubation. The ambulatory group received combined regional nerve block in addition to general anesthesia with endotracheal intubation. Laparoscopic pyeloplasty were performed by the same urological team using a standardized laparoscopic technique. Three 5 mm ports were used, and a double-J stent was routinely placed in both groups. Whether or not to place an abdominal drain was decided according to the intraoperative situation in the inpatient group. Intraoperative factors (e.g., operative time >120 min, bleeding >50 ml, or conversion to open surgery) would disqualify patients from ambulatory discharge. None of these occurred in our cohort.

### Perioperative management

The inpatient group was admitted to the hospital 2 days before the operation to complete preoperative examinations and make preoperative preparations. The preoperative skin was prepared one day before the operation. Fasting and drinking were prohibited for 8 h. Foley catheter was routinely left in place for 3–5 days postoperatively. Postoperative non-steroidal anti-inflammatory drugs (NSAIDs) were given according to the pain scores (FLACC >3 or Facial Expression Score >3) for symptomatic management. After full awakening from general anesthesia (anesthesia awakening score ≥4), a small amount of water was given, along with short-term fluid and electrolyte management. Patients were gradually transitioned to a normal diet for 1–3 days postoperatively, with routine postoperative bed rest and continuous cardiac monitoring for 24 h.

The ambulatory group completed the admission, surgery and discharge within 24 h. Specialist doctors determined the admission group for ambulatory surgery. Patients handled the pre-hospitalization, scanned the WeChat code to join the specialist preparatory medical group (9). Specialist doctors informed the preoperative anesthesia assessment process.

Specialist doctor carried out the online parental classroom education one day before the operation and explains to the family about the operation mode, risks and prognosis knowledge by means of the flipped classroom one day before the surgery.

The patients were prepared for preoperative skin on the day of surgery, prohibited from eating for 6 h (8 h for meat and fried food), and prohibited from drinking for 2 h (6 h for milk). Intraoperative abdominal drains were not placed, and postoperative foley catheters were routinely left in place and removed before discharge. The urinary catheter was not used to manage bladder pressure, as all patients had a double-J stent for ureteral drainage. Early catheter removal was feasible due to stent security.

A small amount of water was given to the patients after they were fully awake from general anesthesia, and they gradually transitioned to a normal diet during the period of hospitalization without supplementation of intravenous nutrition. NSAIDs were routinely used for ultra-early analgesia after they were fully awake from general anesthesia, and were used as appropriate according to the patient's pain condition after that. The patients were routinely monitored by cardiac monitoring for 2 h after the operation, and then got out of bed as early as possible under the guidance of nurses. According to the patient's condition, the appropriate early activity mode was adopted, and the activity time and activity volume were gradually increased.

### Criteria for ambulatory discharges

Specialists and anesthesiologists assessed the patient 3 h after the operation. The criteria for discharge included stable vital signs, clear consciousness, resumption of feeding, no fever, nausea, vomiting, and self-urination after removal of the catheter. After meeting the criteria, the patient was allowed to be discharged from the hospital. Parents were educated about discharge precautions and review time, and were taught to use the Ambulatory Surgery Ward Visiting Guide. Parents were instructed on how to identify complications and management measures, with special attention to cases requiring immediate return to hospital, such as respiratory distress, double J stent blockage and displacement, and urinary tract infection.

#### Postoperative treatment

Both groups were advised to schedule a follow-up one month after surgery, with double J stent removal performed if no abnormalities were detected.

For the ambulatory group, detailed educational materials (texts and videos) regarding preoperative precautions, postoperative care, and home management for hydronephrosis surgery were provided through an official WeChat public account. A personalized patient information database for UPJO was established, and structured telephone follow-ups were conducted on postoperative days 1, 3, 7, and 30. An out-of-hospital safety guarantee system for pediatric ambulatory surgery patients could be activated for emergency support.

### Study parameters

The criteria for successful surgery are no progressive worsening of hydronephrosis on ultrasound, maintenance of stable or sustained improvement in renal function, and no need for reoperation for recurrence of obstruction at 6 months postoperatively.

Perioperative indicators included operative time, hospitalization costs, length of stay, catheter duration, and stent removal time. Postoperative complications included reoperation, stent replacement, urinary tract infection, fever, vomiting, and pain.

According to the Clavien-Dindo Classification System, postoperative complications are categorized. Pain, vomiting, and fever are Grade I. Urinary tract infection (treated with antibiotics) is Grade II. Stent replacement and reoperation are Grade III.

### Statistical analyses

SPSS 29.0 software was used for statistical analysis.

A *post-hoc* power analysis was conducted using GPower 3.1. For hospitalization costs, with an effect size of 1.39, α = 0.05, and sample sizes of 78 and 74, the power exceeded 99%.

According to the results of normality test, the measurements obeying normal distribution were expressed as Mean ± SD, and *t-*test was used for comparison between two groups. The measurements not obeying normal distribution were expressed as median (Q1, Q3), and Mann–Whitney *U*-test was used for comparison between two groups. Categorical variables were expressed as frequencies and percentages, and comparisons between the two groups were made using the *χ*^2^ test. *P* < 0.05 was considered statistically significant. Complication rates were adjusted for multiple comparisons using the Benjamini-Hochberg method [false discovery rate (FDR) < 5%].

## Results

A total of 152 patients with UPJO were employed. 78 patients were included in the inpatient group with a median age of 69 (7, 106) months, a male-to-female ratio of 58:20 (74.4%:25.6%), and a unilateral-to-bilateral ratio of 72:6 (92.3%:7.7%). 74 patients were included in the ambulatory group with a median age of 54 (7, 96.75) months, a male-to-female ratio of 58:16 (78.4%:21.6%) and a unilateral to bilateral ratio of 72:2 (97.3%:2.7%). Age, male to female ratio and unilateral to bilateral ratio were not significantly different between the two groups (Table 1).

**Table 1:** General information and perioperative indicators of children in both groups.

General information and perioperative indicators	Inpatient group (78)	Ambulatory group (74)	*P*
Age (months)	69 (7, 106)	54 (7, 96.75)	0.458^c^
Sex			0.56^b^
Male	58 (74.4%)	58 (78.4%)	
Female	20 (25.6%)	16 (21.6%)	
Unilateral proportion			0.311^b^
Unilateral	72 (92.3%)	72 (97.3%)	
Bilateral	6 (7.7%)	2 (2.7%)	
Operative time (min)	80 (60, 110)	85 (70, 105)	0.1612^c^
Indwelling urinary catheterization duration (days)	4 (3, 5)	1 (1, 1)	0.0001^c^
Length of hospital stay (days)	8 (7, 10.25)	1 (1, 2)	0.0001^c^
Hospitalization costs(yuan)	17,882 ± 2,579	14,291 ± 1,873	0.0001^a^
Postoperative double J stent removal time (days)	36 (32.75, 42)	32.5 (28, 36.25)	0.0016^c^

^a^ Student's *t* test.

^b^ Pearson's *χ*^2^ test.

^c^ *U* test.

In the inpatient group, the median operative time was 80 (60, 110) min. The median length of hospital stay was 8 (7, 10.25) days. The average hospitalization costs were 17,882 ± 2,579 yuan. The median indwelling urinary catheterization duration was 4 (3,5) days. The median postoperative double J stent removal time was 36 (32.75, 42) days. In the ambulatory group the median operative time was 85 (70, 105) min. The median length of hospital stay was 1 (1,2) day. The average hospitalization costs were 14,291 ± 1,873 yuan. The median indwelling urinary catheterization duration was 1 (1,1) day. The median postoperative double J stent removal time was 32.5 (28, 36.25) days. There was no statistical difference in the operative time between the two groups. The length of hospital stay, hospitalization costs, indwelling urinary catheterization duration and postoperative double J stent removal time in the ambulatory group were significantly decreased compared to those in the inpatient group (Table 1).

In the inpatient group, there were 2 cases (2.6%) of reoperation, 2 cases (2.6%) of double J stent replacement, 5 cases (6.4%) of urinary tract infection, 12 cases (15.4%) of vomiting, 3 cases (3.8%) of fever, and 9 cases (11.5%) of pain. In the ambulatory group, there were 2 cases (2.7%) of reoperation, 4 cases (5.4%) of double J stent replacement, and 4 cases (5.4%) of urinary tract infection, vomiting in 12 cases (16.2%), fever in 2 cases (2.7%), and pain in 6 cases (8.1%). There was no statistical difference in the incidence of the above complications between the two groups (Table 2). No intraoperative complications occurred in either group, supporting the feasibility of same-day discharge in uncomplicated cases. Two patients (2.7%) in the ambulatory group required extended hospitalization (>24 h) due to fever and abdominal distension. Unplanned hospital visits within 30 days occurred in 4 patients (5.4%) for urinary tract infection and stent-related issues. Sensitivity analysis adjusting for anesthesia type showed no significant effect on pain incidence (*P* = 0.38).

**Table 2:** Complication rates.

Complications	Inpatient group (78)	Ambulatory group (74)	*P*
Reoperation	2 (2.6%)	2 (2.7%)	1
Double J stent replacement	2 (2.6%)	4 (5.4%)	0.865
Urinary tract infection	5 (6.4%)	4 (5.4%)	1
Vomiting	12 (15.4%)	12 (16.2%)	0.888
Fever	3 (3.8%)	2 (2.7%)	1
Pain	9 (11.5%)	6 (8.1%)	0.860

*P* values adjusted for multiple comparisons via Benjamini-Hochberg procedure (FDR < 5%).

## Discussion

UPJO is a common urological malformation in children. Laparoscopic surgery has become the gold standard in the treatment of hydronephrosis in children due to its comparable efficacy and better cosmetic outcome compared to open surgery (10, 11). Compared with open surgery, laparoscopic surgery requires a more skillful laparoscopic surgeon to shorten the operation time and reduce injuries.

Children undergoing ambulatory surgery have a short hospital stay and need to be discharged after a short postoperative assessment. A longer and more invasive surgery would inevitably lead to an increased risk of discharge. This means that experienced urologists are needed to perform pyeloplasty on ambulatory wards.

It is not common to perform pyeloplasty in ambulatory wards. In addition to the ambulatory pyeloplasty performed at the University of Toronto Children's Hospital (7), cases of children who underwent laparoscopic pyeloplasty using robot-assisted techniques have been reported, involving a total of 32 patients, 26 of whom were successfully discharged on the same day of the operation and 6 others on the following day after the operation. Two children were re-hospitalized within 30 days of surgery for urinary tract infection and double J stent displacement. After a 15-month follow-up, no recurrence was observed in the above patients (12). Our clinic group has accumulated a lot of experience in laparoscopic pyeloplasty and ambulatory ward treatment, and there was no significant difference in the postoperative complication rates between the two groups in this study, indicating that pyeloplasty is safe and effective when performed in an ambulatory ward.

In the case of ambulatory surgery, patients ranging in age from 1 month to 17 years were discharged within 24 h after surgery, suggesting that age is not a major factor in postoperative recovery. Unless there are major complications, there are no uncontrollable factors preventing discharge in the short term after surgery, which reminds us whether routine length-of-stay observation is necessary. One of the more common short-term complications was vomiting, which was considered to be related to the gastrointestinal reaction to anesthesia. Since pyeloplasty does not affect the gastrointestinal tract, the ambulatory group in this study all ate gradually after fully awakening from anesthesia, and completed adequate feedings before discharge, and no recurrent vomiting was observed. The inpatient group followed the traditional concept of waiting for the recovery of gastrointestinal function to gradually increase the diet, which invariably increased medical costs.

The ambulatory group also pioneered the implementation of the enhanced recovery after surgery (ERAS) protocol (Table 3) (13–15), providing preemptive analgesia to patients immediately after anesthesia emergence. Although there was no statistical difference in the number of children with pain, the percentage did decrease. While regional blocks were limited to the ambulatory group, sensitivity analysis ruled out confounding effects on pain outcomes. Both groups had children with postoperative fever. One case in the ambulatory group was treated with oral acetaminophen and returned home for observation, and recovered well after several days. The other case was delayed for one day due to recurrent fever that did not go away, and during the period of hospitalization, the routine blood tests suggested that there might be bacterial infections, so the child was discharged after one day of anti-infective treatment, and was instructed to continue the anti-infective treatment in the outpatient clinic. In addition, there was one case of delayed discharge in the ambulatory group. This child developed mild abdominal distension after surgery, and the postoperative ultrasound suggested an accumulation of fluid in the abdominal cavity, and surgery was proposed to prevent postoperative re-infarction. However, the abdominal distension resolved spontaneously after 3 h of observation, and a repeat ultrasound showed a decrease in peritoneal fluid. Discharge was delayed by one day to ensure safety. The above results remind us that postoperative observation and medical management is an important part of ambulatory surgery. The quality of medical care in the ambulatory ward can be guaranteed by having a specialist to deal with this type of emergency.

**Table 3:** Enhanced recovery after surgery (ERAS) protocol for ambulatory pyeloplasty.

Phase	Intervention
Preoperative	Parental education via WeChat; Fasting: 6 h (solids), 2 h (clear fluids).
Intraoperative	Combined regional anesthesia; No abdominal drains.
Postoperative	Early oral intake; NSAIDs for analgesia; Catheter removal before discharge.

By optimizing the hospitalization process, improving the hospitalization pattern and enhancing the level of medical services, ambulatory surgery reduces the length of hospitalization and medical costs of patients and saves more medical costs while ensuring that patients receive high-quality medical services (16, 17). In order to further ensure the quality of medical care, telephone follow-ups were conducted at 1, 3, 7 and 30 days after the children were discharged from the hospital. This short period of time allows us to understand the changes in the child's condition and provide appropriate guidance, which not only relieves the family's anxiety, but also keeps us abreast of the child's real situation. The success of same-day discharge relied on patient selection, standardized surgical technique, and a safety-focused ERAS pathway. Our data suggest that with these measures, ambulatory LP is safe for uncomplicated UPJO.

The prolonged median LOS of 8 days in the inpatient group primarily reflects adherence to a conventional postoperative pathway, contrasting with the standardized ERAS protocol applied to the ambulatory cohort. Key differences include delayed removal of the indwelling catheter, a more conservative approach to resuming oral intake, and extended monitoring of surgical drains. While medical factors like slower gastrointestinal recovery contributed, the extended LOS was also influenced by deeply ingrained parental expectations in Mainland China favoring prolonged in-hospital observation for pediatric surgery recovery. This cultural preference for cautious convalescence under direct medical supervision, coupled with the absence of a mandated early discharge protocol in the inpatient group, led to the observed discrepancy in LOS and catheter management timelines.

The prognostic success rate of minimally invasive treatment of UPJO ranges from 85% to 100% (18–21). In this study, with a six-month follow-up, the overall success rate is now 94.8% vs. 91.9%, which is not a significant difference. There was also no significant difference between the two groups in terms of double J stent re-tubing and the incidence of urinary tract infections, indicating that minimally invasive UPJO surgery is safe and effective when completed in the ambulatory ward. Of course, it is necessary to constantly adjust and continuously improve to develop an ambulatory surgery management model that meets the level of the medical institution itself.

The non-randomized allocation based on geographic distance is a key limitation. Urban populations in the ambulatory group inherently had advantages such as proximity to emergency services and stronger social support networks, which likely facilitated successful same-day discharge. Conversely, the inpatient group's prolonged hospitalization may reflect logistical necessities rather than clinical need. Although complication rates were comparable between groups, this finding must be interpreted cautiously due to potential unmeasured confounders. Future randomized trials or propensity-score matched studies are essential to validate the safety of ambulatory UPJO surgery across diverse geographic and socioeconomic populations. The generalizability of our results may be constrained by the single-center design and limited sample size. Further multi-center studies are warranted.

In conclusion, minimally invasive surgery for UPJO in ambulatory wards can shorten the length of hospital stay, reduce the risk of nosocomial infections, reduce the burden of accompanying family members, and lower the cost of medical care. However, this model also faces challenges, such as the high demand for short-term postoperative follow-up, the need for close cooperation with parents, and the need for more sophisticated surgeon skills and anesthesia management to ensure the safety and effectiveness of the surgery. Overall, rational application can open up a new path for the treatment of hydronephrosis in infants and children, balancing medical resources and the needs of children.

## Data Availability

The original contributions presented in the study are included in the article/Supplementary Material, further inquiries can be directed to the corresponding author/s.

## References

[1] Jh N. The surgery of infancy. Br Med J. (1909) 2:753–4.

[2] Glowka L Tanella A Hyman JB. Quality indicators and outcomes in ambulatory surgery. Curr Opin Anaesthesiol. (2023) 36(6):624–9. 10.1097/ACO.0000000000001304 37871296

[3] Luo J Xie C Fan D. Historical development and experience of day surgery in China: from the perspective of anesthesiologists. Paediatr Anaesth. (2025) 35(6):412–23. 10.1111/pan.15078 39921339 PMC12060084

[4] Yalcinkaya F Ozcakar ZB. Management of antenatal hydronephrosis. Pediatr Nephrol. (2020) 35(12):2231–9. 10.1007/s00467-019-04420-6 31811536

[5] Cai PY Lee RS. Ureteropelvic junction obstruction/hydronephrosis. Urol Clin North Am. (2023) 50(3):361–9. 10.1016/j.ucl.2023.04.001 37385700

[6] Passoni NM Peters CA. Managing ureteropelvic junction obstruction in the young infant. Front Pediatr. (2020) 8:242. 10.3389/fped.2020.00242 32537441 PMC7267033

[7] Rickard M Chua M Kim JK Keefe DT Milford K Hannick JH Evolving trends in peri-operative management of pediatric ureteropelvic junction obstruction: working towards quicker recovery and day surgery pyeloplasty. World J Urol. (2022) 40(5):1283–4. 10.1007/s00345-021-03925-w 34997295

[8] Vemulakonda VM. Ureteropelvic junction obstruction: diagnosis and management. Curr Opin Pediatr. (2021) 33(2):227–34. 10.1097/MOP.0000000000000994 33470672

[9] Liu H Liu X Lu Y. Use of WeChat applet in the management of ambulatory surgery. Int J Surg. (2023) 109(3):655–7. 10.1097/JS9.0000000000000304 36912523 PMC10389572

[10] Metzelder ML Schier F Petersen C Truss M Ure BM. Laparoscopic transabdominal pyeloplasty in children is feasible irrespective of age. J Urol. (2006) 175(2):688–91. 10.1016/S0022-5347(05)00179-5 16407027

[11] Krajewski W Wojciechowska J Dembowski J Zdrojowy R Szydełko T. Hydronephrosis in the course of ureteropelvic junction obstruction: an underestimated problem? Current opinions on the pathogenesis, diagnosis and treatment. Adv Clin Exp Med. (2017) 26(5):857–64. 10.17219/acem/59509 29068584

[12] Broch A Paye-Jaouen A Bruneau B Glenisson M Taghavi K Botto N Day surgery in children undergoing retroperitoneal robot-assisted laparoscopic pyeloplasty: is it safe and feasible? Eur Urol Open Sci. (2023) 51:55–61. 10.1016/j.euros.2023.03.004 37187722 PMC10175732

[13] Pilkington M Nelson G Pentz B Marchand T Lloyd E Chiu PPL Enhanced recovery after surgery (ERAS) society recommendations for neonatal perioperative care. JAMA Surg. (2024) 159(9):1071–8. 10.1001/jamasurg.2024.2044 39083294

[14] Simpson JC Bao X Agarwala A. Pain management in enhanced recovery after surgery (ERAS) protocols. Clin Colon Rectal Surg. (2019) 32(2):121–8. 10.1055/s-0038-1676477 30833861 PMC6395101

[15] Burgess J Hedrick T. Postoperative analgesia in enhanced recovery after surgery protocols: trends and updates. Am Surg. (2023) 89(2):178–82. 10.1177/00031348221103654 35579300

[16] Hodgson JA Cyr KL Sweitzer B. Patient selection in ambulatory surgery. Best Pract Res Clin Anaesthesiol. (2023) 37(3):357–72. 10.1016/j.bpa.2022.12.005 37938082

[17] Eklund JE Chang CC Donnelly MJ. Critical patient safeguards for ambulatory surgery centers. Curr Opin Anaesthesiol. (2024) 37(6):719–26. 10.1097/ACO.0000000000001434 39377472

[18] Cascini V Lauriti G Di Renzo D Miscia ME Lisi G. Ureteropelvic junction obstruction in infants: open or minimally invasive surgery? A systematic review and meta-analysis. Front Pediatr. (2022) 10:1052440. 10.3389/fped.2022.1052440 36507128 PMC9727311

[19] Autorino R Eden C El-Ghoneimi A Guazzoni G Buffi N Peters CA Robot-assisted and laparoscopic repair of ureteropelvic junction obstruction: a systematic review and meta-analysis. Eur Urol. (2014) 65(2):430–52. 10.1016/j.eururo.2013.06.053 23856037

[20] Gu L Li Y Li X Liu W. Single-port vs multiple-port robot-assisted laparoscopic pyeloplasty for the treatment of ureteropelvic junction obstruction: a systematic review and meta-analysis. J Endourol. (2023) 37(6):681–7. 10.1089/end.2023.0064 37051695

[21] Wang M Xi Y Huang N Wang P Zhang L Zhao M Minimally invasive pyeloplasty versus open pyeloplasty for ureteropelvic junction obstruction in infants: a systematic review and meta-analysis. PeerJ. (2023) 11:e16468. 10.7717/peerj.16468 38025670 PMC10666611

